# Worksite tobacco control – a qualitative study on perspectives from employers and employees at small worksites

**DOI:** 10.1186/s12889-022-13346-y

**Published:** 2022-05-06

**Authors:** Christine M. Kava, Raymond A. Ruiz, Jeffrey R. Harris, Peggy A. Hannon

**Affiliations:** 1 Department of Health Systems and Population Health, University of Washington School of Public Health, Seattle, WA USA; 2 Health Promotion Research Center, University of Washington, 3980 15th Avenue NE, Seattle, WA 98195 USA

**Keywords:** Tobacco, Employees, Employers, Small workplaces, Implementation, Tobacco control, Evidence-based intervention

## Abstract

**Background:**

Evidence-based interventions (EBIs) for tobacco control can be implemented in worksite settings to reduce tobacco use. Small worksites are less likely to adopt tobacco control EBIs than large worksites. The purpose of this qualitative study was to 1) explore factors that impact small employers’ decisions to offer tobacco control EBIs, and 2) understand employees’ perceptions of tobacco control at small worksites.

**Methods:**

Working with staff from small worksites (20–250 employees), we analyzed data from 12 semi-structured interviews with employers (via key informants) and four focus groups with employees. We recruited employers and employees through a purchased business list and market research company, respectively. Interview and focus group topics included perceptions of worksite tobacco control; internal and external forces shaping worksite tobacco control implementation; and perceived worksite support for cessation. We conducted thematic data analysis.

**Results:**

Key themes from the employer interviews included: the local environment played an important role in implementation of tobacco control EBIs; tobacco control was perceived as important but not a priority; and tobacco control decisions were driven by worksite culture. Key themes from the employee focus groups included: perceived employer support for tobacco cessation was limited although there was interest from employees; employees who currently used tobacco were stigmatized for their behavior; and incentives and coaching were considered ideal tobacco control EBIs.

**Conclusions:**

Tobacco control has not been prioritized at small worksites, despite employees welcoming additional cessation support. This study contributes important information on contextual factors and employee preferences that could be targeted to improve tobacco control EBI implementation. Worksites should implement comprehensive tobacco-free policies, minimize stigma when promoting cessation, establish equitable break policies, and involve employees in decision-making related to tobacco control.

**Supplementary Information:**

The online version contains supplementary material available at 10.1186/s12889-022-13346-y.

## Background

Tobacco use and exposure cause several chronic diseases such as cancer, heart disease, and diabetes [1]. Evidence-based interventions (EBIs) for tobacco control reduce chronic-disease risk by increasing cessation rates and offering individuals protection from secondhand smoke [2, 3]. Example EBIs include mobile health text message cessation interventions, smoke-free policies, quitline interventions, reducing out-of-pocket costs for cessation treatment, and incentives and competitions (when combined with additional interventions) [3]. About 60% of U.S. adults are currently employed [4], making the worksite an important venue for tobacco control.

Over 95% of all businesses have fewer than 250 employees, and businesses in the 20–250 size range employ 25% of all workers [5]. Compared to large businesses, small worksites are less likely to adopt EBIs for tobacco control [6, 7]. There is not consensus on what constitutes a “small” worksite. For example, the Small Business Administration commonly defines a small worksite as having fewer than 500 employees [8], while others have defined a small worksite as having fewer than 200 employees [9]. Based on our prior work [10–12], we define a small worksite here as employing 20–250 employees at all locations combined. Recent data from the Workplace Health in America Survey indicate that 70% of worksites with 500 + employees and 15%-28% of companies with 10–249 employees have a policy banning all tobacco use [6]. Similarly, 74% of worksites with 500 + employees and just 16%-35% of worksites with 10–249 employees offer a tobacco cessation program [6].

Small worksites face several challenges to implementing health promotion programs, including tobacco control: greater economic instability, limited financial and personnel resources to support programming, competing priorities, lack of employer confidence or interest in programs in part due to low perceived financial return on investment, and low program participation rates among employees who use tobacco [13–17]. Small worksites also hire a greater proportion of low-wage workers [18, 19], who tend to have higher rates of tobacco use [20, 21], smoke with greater intensity and are less likely to quit smoking [17, 22, 23], and have limited access to health promotion programs [24]. On the other hand, research suggests that small worksites with fewer employees may allow for a more intimate work culture (Goetzel & Ozminkowski, 2008), which could enhance participation in smoking cessation activities, if made available.

Taken together, this information suggests that improving implementation of tobacco control EBIs at small worksites offers a prime opportunity to improve tobacco disparities by increasing assisted quit attempts among low-wage workers. However, much of the literature in this area focuses on larger worksites. Further, while prior studies have examined employers’ views on workplace health promotion [25–27], less has been done to examine perceptions of tobacco control specifically. Additional research is also needed to understand these perspectives among employees, who are critical to EBI implementation. Previous studies have found varied rates of employee participation in EBIs for tobacco control, ranging from as low as 9% to as high as 88% [2]. A better understanding of employees’ perceptions of tobacco control EBIs and worksite support for cessation could help to inform optimal EBI selection, implementation, and promotion strategies to encourage program participation and/or policy compliance.

Building upon prior research [16], the aims of this qualitative study were to 1) explore barriers and facilitators affecting the decisions of employers at small worksites to offer tobacco control EBIs and 2) understand employees’ perceptions and attitudes toward tobacco control and cessation. To answer these aims, we conducted semi-structured interviews and focus groups with key informants representing small worksites (hereafter referred to as employers) as well as employees at small worksites. We hope that our findings can be used to inform strategies to improve tobacco control EBI implementation and participation at small worksites to reduce tobacco-related disparities.

## Methods

### Recruitment and data collection

The study protocol was approved by the University of Washington Institutional Review Board. All interviews and focus groups were conducted via telephone or online over Zoom [28]. We chose to conduct interviews with employers because we were interested in eliciting in-depth information on the tobacco control EBIs at their worksite, including contextual factors that influence implementation. We conducted focus groups with employees for feasibility purposes, and because we were interested in understanding differences in perceptions among employees who used vs. did not use tobacco (described below); the synergistic interactions among participants as they shared their experiences allowed us to yield richer data from an employee perspective.

To be eligible to participate in the study, employers had to be: a) at least 18 years of age; b) employed at a small worksite (20–250 employees across all locations combined); c) responsible for implementing, or knowledgeable about, the tobacco control EBIs at their worksite; and d) working in one of the following states or territories: Arizona, Colorado, District of Columbia, Hawaii, Maryland, Massachusetts, Minnesota, New Jersey, New Mexico, New York, Rhode Island, or Vermont. To obtain a more homogenous sample, we recruited from these states which had similar smoking prevalence and smoke-free indoor-air laws [29, 30].

To recruit employers, we purchased a list of 1,300 businesses from ReferenceUSA [31] based on our eligibility criteria. We removed 72 businesses due to record duplication and/or because we determined that the business employed greater than 250 employees across all locations. We reached out to 1,228 business contacts via e-mail to request their participation in the study. We followed up with non-respondents twice to request their participation. To increase response rates, we followed up with a sample of contacts via telephone. Of the 1,228 individuals we contacted: 1,041 did not respond; 123 could not be reached (e.g., due to e-mail bounces); 33 declined to participate; 14 were deemed ineligible due to business size; 13 agreed to participate; and 4 expressed initial interest in being interviewed but did not respond to further communications. The interviews lasted 30 min on average. We provided a $75 incentive for participation.

We contracted with a market research company (Big Bang Recruiting, Tulsa, OK) to recruit for and schedule the focus groups. Similar to our interviews, to be eligible, focus group participants had to be: a) at least 18 years of age; b) employed at a small worksite; and c) working in one of the states/territories listed above. Participants did not need to have strong knowledge of the tobacco control EBIs at their worksite in order to participate. In total, 315 individuals were screened for focus group participation; of these individuals, 247 were deemed ineligible and 68 qualified to participate, of which 34 were scheduled for a focus group according to their tobacco use. The most common reasons for ineligibility were having a business size smaller than 20 or larger than 250 and/or working outside of one of the states/territories we were recruiting from.

Two of the four focus groups were with employees who had never used tobacco or who had quit more than 12 months ago (i.e., never/former groups). We conducted the other two groups with employees who currently used tobacco or who had quit less than 12 months ago (i.e., current/recent former groups). We grouped participants in this way for two reasons: 1) we wanted to ensure that employees who currently used tobacco felt comfortable sharing their experiences with tobacco use and cessation and 2) given differences in tobacco control perceptions according to tobacco use noted in prior studies [32], we wanted to explore these potential differences in our thematic analysis. We provided a $75 incentive for participation.

### Measures

The employer interview guide (see Additional file 1) contained 11 open-ended questions informed by the Consolidated Framework for Implementation Research [33] and Pettigrew’s framework on receptive contexts for change [34]. We included questions on the following topics: a) background information, including job and worksite characteristics; b) tobacco EBIs offered at the worksite; c) external forces shaping tobacco control at the worksite, including the impact of COVID-19 on tobacco control perceptions (outer setting); d) internal forces shaping worksite tobacco control (inner setting); and e) decision-making processes for tobacco control. At the end of the interview, we collected information on participants' age, gender, race, ethnicity, and smoking status.

The focus group guide (see Additional file 2) contained eight questions on the following topics: a) tobacco interventions offered at the worksite; b) perceptions of worksite tobacco control, including the impact of COVID-19 on tobacco control; c) perceived norms around tobacco; d) cessation experiences, including resources used to quit and worksite support for cessation (asked in current/recent former groups only); and e) opinions on what an ideal tobacco intervention would look like. When scheduling for the focus groups, the market research company collected information on demographics and tobacco use.

### Data analysis

We conducted data analysis in Atlas.ti version 8 [35]. We followed the same analytic approach for the interviews and focus groups. First, we created codebooks based on the interview and focus group questions. Then, two members of the research team double-coded a portion of the transcripts and came together to discuss agreement in coding. For the interviews, the first author (CMK) coded all transcripts with the final codebook. For the focus groups, CMK and the second author (RAR) each coded two transcripts with the final codebook. To identify themes and sub-themes, we conducted a careful read of the code reports and created a case-ordered descriptive-matrix display based on guidelines from Miles and Huberman [36].

## Results

In total, we conducted 13 semi-structured interviews with 14 employers and four focus groups with 29 employees. We excluded one employer from our analysis after it was discovered during the course of the interview that they worked for a company employing over 250 employees; thus, the findings reported here are based on 12 interviews with 13 employers. The mean age of employers (Table 1) was 53 years (SD = 11). The largest proportions of employers were male (54%), White (92%), and Non-Hispanic (100%). Sixty-two percent had never smoked, 38% had formerly smoked, and no employers currently smoked. All employers indicated that their worksite offered health insurance to employees. The mean age of employees (Table 2) in the focus groups was 42 years (SD = 14). The largest proportions of employees were female (59%), White (76%), and Non-Hispanic (83%). About 38% of employees currently used tobacco, followed by 35% who formerly used tobacco and 28% who never used.

**Table 1:** Participant characteristics, employers, *n* = 13

Variable	n	%
Gender		
Male	7	54
Female	6	46
Race		
White	12	92
Asian	1	8
Black or African American	0	0
Multiracial	0	0
Ethnicity		
Non-Hispanic	13	100
Hispanic	0	0
Cigarette use		
Never smoked	8	62
Former cigarette use	5	38
Current cigarette use	0	0
Job title		
Executive Director	4	31
HR Director/Consultant	3	23
Owner	2	15
President	2	15
Chief Operating Officer	1	8
Medical Assistant	1	8
Worksite standard industry classification		
Services	5	38
Manufacturing	2	15
Retail Trade	3	23
Construction	1	8
Finance, Industry, Real Estate	1	8
Public Administration	1	8
Worksite state		
Vermont	5	38
Massachusetts	2	15
Minnesota	2	15
New York	2	15
New Jersey	1	8
Rhode Island	1	8

Percentages may not equal 100% due to rounding

**Table 2:** Participant characteristics, employees, *n* = 29

Variable	n	%
Gender		
Male	12	41
Female	17	59
Race		
White	22	76
Asian	3	10
Black or African American	3	10
Multiracial	1	3
Ethnicity		
Non-Hispanic	24	83
Hispanic	5	17
Tobacco Use		
Current tobacco use	11	38
Never used tobacco	8	28
Former tobacco use quit > 12 months ago	6	21
Former tobacco use quit < 12 months ago	4	14
Intention to quit within next six months^a^		
Yes	4	40
No	3	30
No sure	3	30
Education level		
College graduate	18	62
Some college or technical school	9	31
High school graduate	2	7
Annual household income		
Less than $100,000	17	61
$100,000 or more	11	39
Worksite state		
New Jersey	7	24
Massachusetts	5	17
Colorado	4	14
New York	4	14
Arizona	3	10
New Mexico	2	7
Rhode Island	2	7
Hawaii	1	3
Maryland	1	3

Percentages may not equal 100% due to rounding and missing data.

^a^Asked only among employees who currently used tobacco

### Employer Interviews

Three key themes arose from our interviews: 1) the local environment played an important role in tobacco control EBI implementation; 2) tobacco control was perceived as important but not a priority; and 3) decisions made about tobacco control were driven by worksite culture. Each of these themes is described in detail below.

#### Theme 1: The Local Environment Shaped Tobacco Control

Participants described several features of the local environment that facilitated their worksite’s tobacco control efforts. A common facilitator was external policies, such as clean-indoor-air laws. In some cases, enactment of these laws pushed worksites to develop an internal policy or allowed for a more proactive approach to enforcement of, and compliance with, current policies. State political climate also had an impact, with participants in more liberal states noting that people tended to be more supportive and accepting of tobacco control efforts:

*“So, I think people in [state] are a little bit more aware about the rules and regulations and are a little less pro-smoking than some other areas I’ve been to. The state laws help, the local laws help.”* – Male, 45, Former Cigarette Use

A few participants described collaborating with local agencies on tobacco control interventions, and that this made implementation easier:

*“I mean the [external agency collaboration was] huge…even to the costs of implementing, not just creating the policy and understanding best practices and how we're going to enforce things, but the actual cost of implementation was burdened on somebody else and not on our organization. So that's certainly helpful.”* – Male, 42, Never Smoked

Of note, when we asked participants to describe how local competitors influenced their decisions around tobacco control, nearly all indicated that competitors did not influence implementation.

#### Theme 2: Tobacco Control Important but Not Prioritized

While participants were supportive of tobacco control efforts, most indicated that it was not a priority at their worksite. In many cases, participants felt like there were not enough employees who used tobacco or issues with compliance to warrant making tobacco control a high priority:

*“I don't think [tobacco control EBIs have] been prioritized enough, but it may have to do with the fact that we don't have that many smokers…we’re geared more towards overall healthy lifestyle...I think we focus on that a little bit more because we have more staff who need help with it.”* – Male, 45, Former Cigarette Use

Participants highlighted tobacco use as being lower and more stigmatized now than in the past, and noted that employees who did use tobacco tended to hide their behavior or do it away from others. In other cases, participants described being satisfied with interventions already in place. While COVID-19 did not have a direct impact on tobacco control efforts at most worksites, a couple of participants described tobacco control dropping in priority due to more pressing issues that had emerged since the pandemic began.

Some participants described being open to implementing new interventions if a demonstrated need among employees existed. When we asked participants to describe key attributes that they would look for when deciding whether or not to adopt a new tobacco control intervention, a few participants described the value in having a prescribed or “turn-key” program to make implementation easier (e.g., ready-made resources, draft policy language, etc.). A couple of participants described the importance of understanding the context for tobacco use and cessation, specifically motivations for use, and the extent to which employees are interested in engaging with tobacco cessation programs. Otherwise, attributes described as desirable varied substantially and included: tailored to the worksite’s industry; evidence-based; has the capability to measure work impact; contains inclusive materials and messaging (e.g., avoids stigmatizing individuals with disabilities caused by tobacco use); and has good graphic design for cessation resources (e.g., posters).

#### Theme 3: Decision-Making Driven by Company Culture

The culture of the worksite, including leadership support, drove decisions made about tobacco control. Several participants described having a worksite culture or mission aligned with health and wellness; it was made clear by some that tobacco use was not consistent with this culture:

*“Well, I mean, we work with young children and so we talk a lot about secondhand smoke and the impact that that has on families…the fact that we work with very young, tender lives is what influences us most to promote anti-tobacco you know, promote not [using] tobacco products.”* – Female, 65, Former Cigarette Use

A couple of participants described lacking management support for tobacco-free policies in the past, with subsequent changes in leadership helping to improve enforcement and compliance measures:

*“Well it used to be not so good, because two of our managers smoked, and so they would take employees out on the curb across the street and have a smoke break…And now none of our managers smoke and they're a little more mature, some of them have families, and so I think that makes a huge difference because there's no one, at least in our businesses that foster smoking anymore.”* – Male, 64, Never Smoked

One participant, an executive director, described his experience using tobacco and being exposed to secondhand smoke in his prior job, and said this influenced his decision to implement tobacco control interventions at his current worksite.

When we asked participants how their worksite makes decisions about whether to adopt a new tobacco control intervention, senior leadership was described as being at the forefront of decision making. In some cases, leaders sought feedback and/or approval from an advisory board. Though not all leaders sought feedback from employees, those that did described communicating with employees during monthly staff meetings and via informal conversations. In one case, the participant reached out to the one employee who smoked at their worksite to see if a new tobacco policy would present any issues for that individual. Participants also described receiving input from supervisors, who helped to inform decision-making and gauge buy-in from employees.

### Employee Focus Groups

Three key themes arose from our focus groups: 1) employer support for tobacco cessation was limited although there was interest from employees; 2) employees who used tobacco were stigmatized for their behavior; and 3) incentives and coaching were considered ideal interventions. These themes are described in detail below.

#### Theme 1: Employer Support for Tobacco Cessation was Limited Despite Interest from Employees

Tobacco control EBIs were primarily limited to tobacco-free policies and provision of health insurance benefits for cessation:

*“In my case, it’s you’re not allowed to smoke at work. You can smoke at about 50-100 feet away actually from the premises, but I don’t expect them honestly to offer promotions to quit smoking. It’s just kind of up to you. I think all they care about is you come to work; you show up on time and you do as you’re told. You be respectful to other employees and be respectful to your boss. You meet your quota and be respectful and do what’s being asked of you, and go home having done what you’re supposed to do. That’s really all that my work cares about. I don’t think that they care a whole lot about trying to get employees to quit smoking.”* – Male, 42, Current Tobacco Use

Most employees described their worksites as having a no-indoor-smoking policy. In many cases, tobacco use was also prohibited on the premises, or there were restrictions on outdoor use (e.g., tobacco use prohibited within 50 feet from building entrances). Participants also described specific benefits offered through their health insurance (e.g., access to nicotine replacement therapy), although these were sometimes perceived as cessation programs external to their worksite. These and other programs mentioned were often described as under-promoted at their worksite and under-utilized by its employees. Some participants felt it was not the responsibility of their worksite to offer support for cessation, although perceptions of tobacco control EBIs were generally positive:

*“I think it’s great that companies are encouraging people to be smoke-free and giving them the support. It’s not just telling them, “Hey, we’re not going to be—we have a smoke-free environment and just expect them to adapt I guess without the supportive services like the previous folks had mentioned with the smoking cessation programs. I think it’s helpful, too, if you’re going to evoke a smoke-free environment to assist with the supportive services necessary to do so.”* – Female, 35, Never Tobacco Use

#### Theme 2: Employees Who Used Tobacco Stigmatized for Their Behavior

Participants who currently used tobacco discussed hiding their behavior from co-workers due to the stigma associated with tobacco use. For example, some participants described not using tobacco while at work or leaving their worksite to use tobacco (e.g., in their car parked away from building), wearing perfume to cover up the smell of tobacco smoke, or using other tobacco products like smokeless tobacco or e-cigarettes to be more discreet:

*“So, in my work I think that everybody is pretty much healthy. There’s only a few of us who smoke. So, if we do decide to smoke, most of the time we will just like get in the car and then park a few blocks away. And then we smoke and then we go back again, but we would spray perfume all over our bodies so that not a lot of people could smell it. Just because it’s a co-working space so if you stink, anybody could just smell it right away.”* – Female, 27, Current Tobacco Use

One employee described not disclosing their smoking status in order to obtain jobs because they believed they would not be hired otherwise. Relatedly, some participants who did not currently use tobacco described those who did getting more breaks at work, which was perceived as unfair:

*“…smokers get a lot more breaks than non-smokers. So, a bunch of non-smokers took up, “Hey, can we get an extra week of vacation because we don’t take smoke breaks?” So just something similar like that, it kind of sounds selfish, but at the same time they’re going to the effort for smokers, you know? You kind of feel a little not betrayed, but a little slighted if you’re a non-smoker.”* – Male, 31, Never Tobacco Use

#### Theme 3: Incentives and Coaching Considered Ideal Interventions

When we asked employees to describe what an ideal tobacco control EBI would look like at their worksite, several employees mentioned offering incentives for being tobacco-free as well as cessation coaching or counseling. Example incentives included money, gift cards, and lowered healthcare cost-sharing (e.g., reduced monthly premiums):

*“I would tend to think an incentive also, like give me a reason why I should quit. I know that’s the addiction that’s speaking out that way. I mean, it could be money-wise, or it could be gift cards or anything to that effect. I think that an incentive would be very powerful with a lot of people — not everybody, but quite a few people.”* – Female, 52, Current Tobacco Use

A few employees described wanting a designated worksite space for cessation coaching and flexibility to access these services during the workday:

*“…we offer one-on-one coaching through our employee health insurance. I noticed that a lot of their meeting availability for coaches is during the day, Monday-Friday, which is typically when everybody is at work. So maybe some flexibility there and maybe having a designated room or a designated office where those employees could go and meet with their coach virtually.” –* Female, 37, Former Tobacco Use

Programs mentioned less frequently included a progressive shift toward 100% tobacco-free policies, social support from co-workers to quit, and mobile phone apps for cessation.

When asked about how to best promote these interventions, employees described e-mail as the preferred method. E-mail was identified as an ideal promotion method because it was used on a daily basis by many employees and in some cases already being used to promote other worksite programs. Additional promotion methods included posting via the worksite’s Intranet site and in common areas. Some participants favored incorporating cessation support into broader health promotion programming. Regardless of program offered, participants described success being dependent on employees’ internal motivation to quit. Some participants who did not currently use tobacco expressed a desire that similar incentives be offered for other health behaviors (e.g., physical activity), and a few participants described existing wellness programs at their worksite that offered such incentives (e.g., reimbursement for gym memberships).

#### Additional Themes

Two additional themes arose from our analysis of the focus group data: 1) e-cigarettes and vaping were not seen as a healthy alternative to cigarette smoking and 2) the COVID-19 pandemic has influenced tobacco use. Several employees, both who used and did not use tobacco, believed that e-cigarettes should be included in tobacco-free policies and that they did not help with smoking cessation. Some participants described e-cigarettes as being more convenient and easier to conceal, and that this allowed employees to use tobacco in smoke-free locations. As such, e-cigarettes were perceived as being more addictive than cigarettes and leading to more tobacco use overall:

“*I’ve had friends switch to e-cigarettes, because like someone said that they don’t smell. They don’t stink after they smoke and so it didn’t help them at all. It helped them not smell bad and it’s not noticeable that they’re smoking sometimes. But then yes, if anything, they’re smoking more and taking in more nicotine than they were before probably.”* – Male, 31, Never Tobacco Use

*“I think that more people are smoking because of e-cigarettes. It’s the convenience, you know? You turn it on and don’t have to finish the whole cigarette and just take two puffs, take five puffs. I notice a lot more people with their little pens running around.”* – Female, 37, Former Tobacco Use

However, some participants believed e-cigarettes could help with cessation if a stepdown approach (e.g., consuming higher to lower amounts of nicotine) was taken. Participants also described e-cigarette use as being more socially acceptable than combustible cigarettes and being used most among younger employees.

Regarding COVID-19, some participants described the pandemic as contributing to an increase in tobacco use, in part due to increased stress and ease of using tobacco while working from home:

*“Because labor is one of the few things they can control, so people that are still there are given I think a much heavier workload in some professions. A lot of people smoke to cope with the stress. I could see how that would equate to people continuing to smoke or smoking more than they may have prior to COVID.”* – Female, 57, Current Tobacco Use

*“I think with people working from home, it’s easier for people to smoke on the job I guess. I mean, I even see people when we’re having Zoom meetings, they’ll be hitting their vape which normally if we were in the office, you know, that wouldn't be a thing…I think that it’s a game changer for people as far as the amount that they could smoke throughout their workday.”* – Female, 35, Current Tobacco Use

One participant described switching from cigarettes to smokeless tobacco, since they were working from home and their roommates did not like the smell of smoke. The extent to which the pandemic influenced worksite tobacco control was less clear. A few participants described both positive and negative changes in their worksite’s practices overall, including a greater push toward wellness, cuts to health benefits, and company downsizing. Opinions of tobacco use and worksite tobacco control EBIs seemed relatively unchanged due to the pandemic.

## Discussion

The purpose of this qualitative study was to explore contextual factors that influence worksite tobacco control. Our study was guided by the Consolidated Framework for Implementation Research [33] and Pettigrew’s framework on receptive contexts for change [34], both of which describe inner- and outer-setting characteristics that can influence implementation. Our study addresses gaps in the literature by focusing on an understudied setting (small worksites) and includes perspectives from employers and employees, both of whom are critical to successful EBI implementation.

Inner-setting characteristics like organizational culture and individual knowledge and beliefs about tobacco control strongly influenced implementation of tobacco control EBIs. Participants were supportive of tobacco control but indicated that it was not highly prioritized at their worksite. Employers expressed satisfaction with the interventions currently in place and cited a low prevalence of tobacco use within their company as reasons their worksite chose not to prioritize tobacco control.

On the other hand, while some employees did not think it was the responsibility of their employer to offer cessation support, most supported tobacco control efforts at their worksite, expressed a desire for greater cessation assistance, and believed that current cessation support offered was under-promoted and under-utilized. Our findings are consistent with prior studies reporting differences in worksite wellness perceptions between employers and employees [27, 37], and suggest that opportunities exist to improve tobacco control EBI implementation and promotion at small worksites.

As noted earlier, the Guide to Community Preventive Services recommends several EBIs for reducing tobacco use that could be implemented or promoted at the worksite, including mobile health text message cessation interventions, smoke-free policies, quitline interventions, reducing out-of-pocket costs for cessation treatment, and incentives and competitions (when combined with additional interventions) [3]. Many of these recommendations align with what employees described as “ideal” interventions, including incentives and counseling.

Related to policy, several employees did not think that e-cigarettes helped with smoking cessation and were generally supportive of restricting their use at the worksite. This is consistent with a recent study examining attitudes toward e-cigarette workplace policies, in which nearly one third of employees supported such a policy [38]. Given employees’ negative attitudes toward e-cigarettes, as well as evidence to suggest that the aerosol produced by e-cigarette use releases toxins into the environment [39], we see clear opportunities at the state, local, and organizational levels to implement 100% tobacco-free policies. Future studies are needed to explore perceptions of e-cigarette use as a cessation aid among employees.

Both employers and employees described stigma associated with tobacco use. Given these findings, EBIs should be promoted in a way that attempts to minimize feelings of being “called out” by, for example, incorporating messages about tobacco cessation into broader wellness communications sent to all employees. In doing so, employees may be more willing to utilize the cessation supports offered at their worksite, as prior studies have suggested that quit intentions and interest are lower in the presence of potential stigma [40, 41]. Similar to past studies [42], participants of our study described tensions at work due to employees who use tobacco receiving more break time. Establishing equitable break policies at the worksite could help to reduce tensions between employees who do and do not use tobacco, and in turn further reduce stigma and increase employees’ perceptions of cessation support.

Employers described several factors they would consider when deciding whether to adopt a new tobacco control EBI, including a better understanding of employees’ tobacco use and interest in cessation support. Some employers described a low prevalence of tobacco use at their worksite, but given that several employees described hiding their tobacco use from others, the true prevalence may be higher than perceived. While employers described leaders as being the primary decision-makers for tobacco control, eliciting feedback directly from employees could help to ensure that any EBIs being adopted or improved upon address the needs of the workforce.

Related to the outer setting, employers described several environmental features that assisted with EBI implementation. Notably, external policies and incentives like statewide clean-indoor-air laws necessitated increased EBI implementation, and in some cases, pushed worksites to develop more comprehensive internal policies. As alluded to earlier, while many states and localities have policies that restrict tobacco use at the worksite, there is variation in coverage across the U.S. [43]. Enacting comprehensive tobacco-free policies at the state and local levels would increase environmental support for quitting at small worksites.

Lastly, some participants described COVID-19 causing a shift in worksite priorities and in some cases away from tobacco control, despite perceptions that tobacco use had increased during the pandemic. Given recent studies suggesting worse COVID-19 progression among people who smoke [44, 45], demonstrating a connection between greater cessation support and improved worksite operations might encourage employers to consider tobacco control a higher priority. However, future studies are needed to assess the extent to which tobacco use during the pandemic has impacted worksites. Understanding how emergent priorities like COVID-19 influence tobacco control could help inform strategies to address barriers to EBI implementation.

This study should be considered in light of several strengths and limitations. A strength is that we collected data from both employers and employees, and this provided us with a greater understanding of the context for tobacco control at small worksites and allowed us to compare the perspectives of both groups. We also collected data from diverse worksite industries, thus our findings may be relevant to a broader group of small worksites. A limitation is that we did not speak with participants from each of the 12 states or territories we attempted to recruit from. Second, there may be important differences among those who participated in the study and those who declined or did not respond; for example, participants may be more supportive of tobacco control efforts overall. Lastly, the education and income levels of employees who participated in the focus groups were relatively high; the opinions of these participants may therefore differ from lower-wage employees. Despite these limitations, our study provides important insight into tobacco control EBI implementation at small worksites.

## Conclusion

Improving implementation of tobacco control EBIs at small worksites offers a prime opportunity to improve tobacco disparities. Exploring employers’ and employees’ perceptions is important for contextual understanding of the tobacco control environment, including barriers and facilitators to implementation. Our results showed that participants supported tobacco control but did not perceive it to be a high worksite priority. However, employees were generally positive about more tobacco control at their worksites and offered specific preferences for EBIs they perceived as most helpful. This study suggests opportunities to improve tobacco control EBI implementation and promotion at small worksites. Based on our findings, worksites should consider implementing comprehensive tobacco-free policies, promoting tobacco cessation in ways that minimize stigma, establishing equitable break policies, and involving employees when making decisions about what EBIs to adopt. In doing so, negative consequences associated with tobacco use and exposure can be reduced.

## Supplementary Information

**Additional file 1.** Interview Guide. Key informant interview guide.

**Additional file 2.** Focus Group Guide. Employee focus group questions.

## Data Availability

The data underlying this article cannot be shared publicly in order to protect the privacy of individuals that participated in the study. The data will be shared upon reasonable request to the corresponding author.

## Abbreviations

EBI: Evidence-based intervention
SD: Standard deviation
