# Supplementary material for: Worksite tobacco control – a qualitative study on perspectives from employers and employees at small worksites
Source: BMC Public Health. 2022 May 6;22:904. doi: 10.1186/s12889-022-13346-y (PMC9073486; doi:10.1186/s12889-022-13346-y)
Supplement: Supplementary file 1 — Additional file 1. Interview Guide. Key informant interview guide. [file 12889_2022_13346_MOESM1_ESM.docx]

**Interview Guide**

# Background

1. Describe your job position and responsibilities.
   1. What does a typical day look like for you?
2. Tell me about your worksite.
   1. Who are the typical customers and/or clients that your worksite serves?
   2. Does your worksite offer health insurance to most employees?

# Worksite Tobacco Programs

Tobacco programs can be defined as any policy or resource offered to help employees quit using tobacco and/or reduce employees’ exposure to secondhand smoke or aerosols. Example programs that have proven effective include policies that limit tobacco use at the worksite, promoting quit-line interventions, and offering tobacco-cessation-treatment benefits through employer-sponsored health plans.

1. Describe the tobacco programs at your worksite.
   1. Is tobacco use prohibited or limited to certain areas?
   2. How are the programs promoted and/or enforced?
   3. Do any of the programs cover e-cigarettes or other alternative tobacco products? If no, why not?
2. How have forces outside of your worksite, such as laws or mandates, expert recommendations, or guidelines for your industry, affected the tobacco programs at your worksite? Have any of these (or other issues) changed how you think about worksite tobacco programs?
3. Have other worksites (e.g. competitors) influenced your own worksite’s decision to offer tobacco programs? If so, how?
4. What features of the local environment or community have helped your worksite implement tobacco programs? What features of your local environment or community have made implementation harder?
5. Has the COVID-19 pandemic impacted your worksite’s ability to implement tobacco programs? If so, how?
   1. How has the pandemic changed your perceptions about worksite tobacco programs?
6. What features or characteristics of your worksite, including workforce characteristics, have helped you to implement tobacco programs? What characteristics have made implementation harder?
   1. How supportive is your worksite’s leadership of tobacco programs?
   2. How do employees feel about tobacco use? Tobacco programs?
   3. How well does your organization’s culture (e.g. norms, values, assumptions) align with efforts to implement tobacco programs?
   4. Compared to other high-priority initiatives, how have tobacco programs been prioritized?
7. Within your organization, what resources or types of support do you feel are necessary to implement worksite tobacco control programs?
   1. Which of these does your worksite currently have? Which are limited or unavailable?
8. How does your worksite make decisions about whether to adopt a new tobacco program?
   1. Are there key attributes that you look for in a program? *If respondent requests clarification: For example, whether the program is internally or externally developed, the quality of evidence demonstrating its effectiveness, its relative advantage to other programs, how well the program can be adapted to meet the needs of your worksite, and program costs*.
   2. How is feedback from different groups (e.g. managers, supervisors, employees) incorporated into the decision-making process?
   3. Once a program is chosen, who is typically involved in the implementation process? How are decisions about implementation communicated to different groups?

# Closing Questions

1. Is there anything else you would like to share with me today?
2. What is your age? _____
3. What is your gender?
   1. Male
   2. Female
   3. Other identity: __________
4. What is your race? __________
5. Do you identify as Hispanic or Latino/a?
   1. Yes
   2. No
6. Are you a current or former smoker?
   1. Yes – current smoker
   2. Yes – former smoker
   3. No
7. What address should we send the $75 incentive to?
