# Supplementary material for: Worksite tobacco control – a qualitative study on perspectives from employers and employees at small worksites
Source: BMC Public Health. 2022 May 6;22:904. doi: 10.1186/s12889-022-13346-y (PMC9073486; doi:10.1186/s12889-022-13346-y)
Supplement: Supplementary file 2 — Additional file 2. Focus Group Guide. Employee focus group questions. [file 12889_2022_13346_MOESM2_ESM.docx]

**Focus Group Guide**

Worksite tobacco programs can be defined as any policy or resource offered to help employees quit using tobacco and/or reduce employees’ exposure to secondhand smoke or aerosols. Examples include policies that limit tobacco use at the worksite, tobacco-cessation-treatment benefits offered through employer health insurance, and worksite promotion of resources to help quit tobacco (e.g., quit-line).

1. What tobacco programs does your worksite offer?
2. In general, what do you think about worksite tobacco programs?
3. Has the COVID-19 pandemic changed your perceptions of tobacco use or worksite tobacco programs? If so, how?
4. Describe the norms around tobacco use at your worksite.
   1. What do you think about these norms?
   2. What are the norms around e-cigarettes? Are these norms the same or different from other forms of tobacco use?
5. Should worksite tobacco-free policies include e-cigarettes? Can you tell me more about your opinion?
   1. To what extent do you think e-cigarettes help employees quit smoking?
6. *[Current/recent former tobacco groups only]:* What resources (e.g., cessation medications, counseling, self-help materials), if any, have you tried to help you quit using tobacco?
   1. Of the resources you just described, which came from your worksite?
   2. To what extent do you feel like your worksite has supported or currently supports your cessation efforts?
   3. Is there anything your worksite has done or currently does to make quitting harder?
   4. *[If not addressed from responses above]:* How have tobacco-free policies at your worksite influenced your tobacco use?
7. If you were to design an ideal tobacco program for your worksite, what would it look like?
   1. How should the worksite promote this program to employees?
8. Is there anything else you would to share with me today?
